# Robot-assisted total hip arthroplasty: does improved precision translate into better clinical outcomes? A systematic review

**DOI:** 10.1007/s00264-026-07052-2

**Published:** 2026-10-03

**Authors:** Ernesto Muñoz-Mahamud, Miquel Cortés-Rovira, Alfonso Alías-Petralanda, Jenaro-Angel Fernández-Valencia, Andrés Combalia

**Affiliations:** 1 Departament de Cirurgia i Especialitats Medicoquirúrgiques, Facultat de Medicina i Ciències de la Salut, Universitat de Barcelona (UB), c. Casanova, 143, 08036 Barcelona, Spain; 2 Department Orthopaedic Surgery and Traumatology, Hospital Clínic de Barcelona, Barcelona, Spain; 3 Fundació de Recerca Clínic Barcelona-Institut d’Investigacions Biomèdiques August Pi i Sunyer, Barcelona, Spain

**Keywords:** Total hip arthroplasty, Robot-assisted surgery, Robotics, Component positioning, Functional outcomes, Systematic review

## Abstract

**Purpose:**

Robot-assisted total hip arthroplasty (R-THA) is being adopted progressively because of its potential to improve implant positioning and restore hip biomechanics more accurately than conventional techniques. However, whether these technical advantages translate into meaningful clinical benefits remains uncertain. This systematic review compared robot-assisted and conventional THA regarding implant positioning, restoration of hip biomechanics, functional outcomes, and complications.

**Materials and methods:**

A systematic review was performed according to the PRISMA guidelines. PubMed and Scopus were searched for comparative studies published between 2015 and September 2025 evaluating robot-assisted versus conventional THA. After applying predefined eligibility criteria, 13 studies were included. Methodological quality was assessed using the MINORS and RoB 2 tools, and findings were synthesized qualitatively.

**Results:**

Robot-assisted THA was consistently associated with greater accuracy of acetabular component placement within established safe zones and more precise restoration of limb length. Functional outcomes were inconsistent across studies, with no clear evidence of superior patient-reported outcomes or overall clinical benefit. Several studies reported lower rates of dislocation and reoperation following robot-assisted THA, although differences in overall complication rates were less consistent.

**Conclusions:**

Robot-assisted THA improves the accuracy of implant positioning and better reproduces the preoperative surgical plan than conventional THA. However, these technical advantages have not translated into superior functional outcomes in the short to medium term. Longer-term studies are needed to determine their impact on implant survival.

## Introduction

Total hip arthroplasty (THA) is one of the most successful orthopaedic procedures, providing reliable pain relief, restoration of function, and improved quality of life for patients with end-stage hip disease. As the demand for THA continues to increase worldwide, improving surgical accuracy and implant longevity has become a major focus of innovation in hip arthroplasty [1–3].

Robot-assisted THA has emerged as an alternative to conventional techniques by integrating three-dimensional preoperative planning with intraoperative guidance to improve the accuracy and reproducibility of implant positioning. Accurate restoration of acetabular orientation, hip biomechanics, and limb length is considered essential to optimize joint stability and potentially reduce complications such as dislocation, accelerated wear, and leg length discrepancy [4, 5].

Over the past decade, several comparative studies have evaluated whether the improved precision achieved with robotic systems translates into better clinical outcomes. Although robot-assisted THA has been associated with greater accuracy in component positioning and closer adherence to predefined safe zones, evidence regarding improvements in patient-reported outcomes, functional recovery, complications, and implant survivorship remains inconsistent [6, 7].

Therefore, the aim of this systematic review was to evaluate whether the greater precision achieved with robot-assisted total hip arthroplasty translates into clinically meaningful improvements in patient outcomes by comparing implant positioning accuracy, functional outcomes, and postoperative complications between robot-assisted and conventional total hip arthroplasty.

## Material and methods

This systematic review was conducted in accordance with the PRISMA 2020 guidelines. The review question was formulated using the PICO framework to compare robot-assisted total hip arthroplasty (R-THA) with conventional total hip arthroplasty (C-THA) regarding implant positioning accuracy, functional outcomes, and postoperative complications.

### Search strategy

A systematic literature search was performed in the PubMed and Scopus databases to identify comparative studies published between January 2015 and September 2025. The search combined Medical Subject Headings (MeSH) and free-text terms related to total hip arthroplasty, robotic surgery, implant positioning, and clinical outcomes. The final search strategy was: ("Arthroplasty, Replacement, Hip"[MeSH] OR "Total Hip Replacement" OR "THA") AND ("Robotic Surgical Procedures"[MeSH] OR "robot-assisted surgery" OR "robotic hip arthroplasty" OR "MAKO") AND ("Treatment Outcome"[MeSH] OR "accuracy" OR "component positioning" OR "implant positioning" OR "cup placement" OR "anteversion" OR "inclination" OR "complications" OR "revision" OR "dislocation" OR "periprosthetic fracture"). In addition, the reference lists of the included studies were manually screened to identify any additional eligible articles.

### Eligibility criteria

Studies were considered eligible if they: (1) directly compared robot-assisted and conventional THA; (2) included adult patients undergoing primary THA; (3) had a randomized controlled trial, prospective cohort, retrospective cohort, or case–control design; (4) reported quantitative data on at least one of the following outcomes: implant positioning accuracy, functional outcomes, or postoperative complications; and (5) had a minimum follow-up of three months.

Studies were excluded if they were cadaveric, animal, biomechanical, or simulation studies; systematic reviews, meta-analyses, narrative reviews, editorials, or expert opinions; studies involving pediatric patients; studies without a comparative conventional THA group or lacking quantitative outcome data; articles published before 2015 or in languages other than English; and studies focusing exclusively on highly selected patient populations that were not representative of routine clinical practice. Articles for which the full text was unavailable were also excluded.

### Study selection

**Fig. 1:**
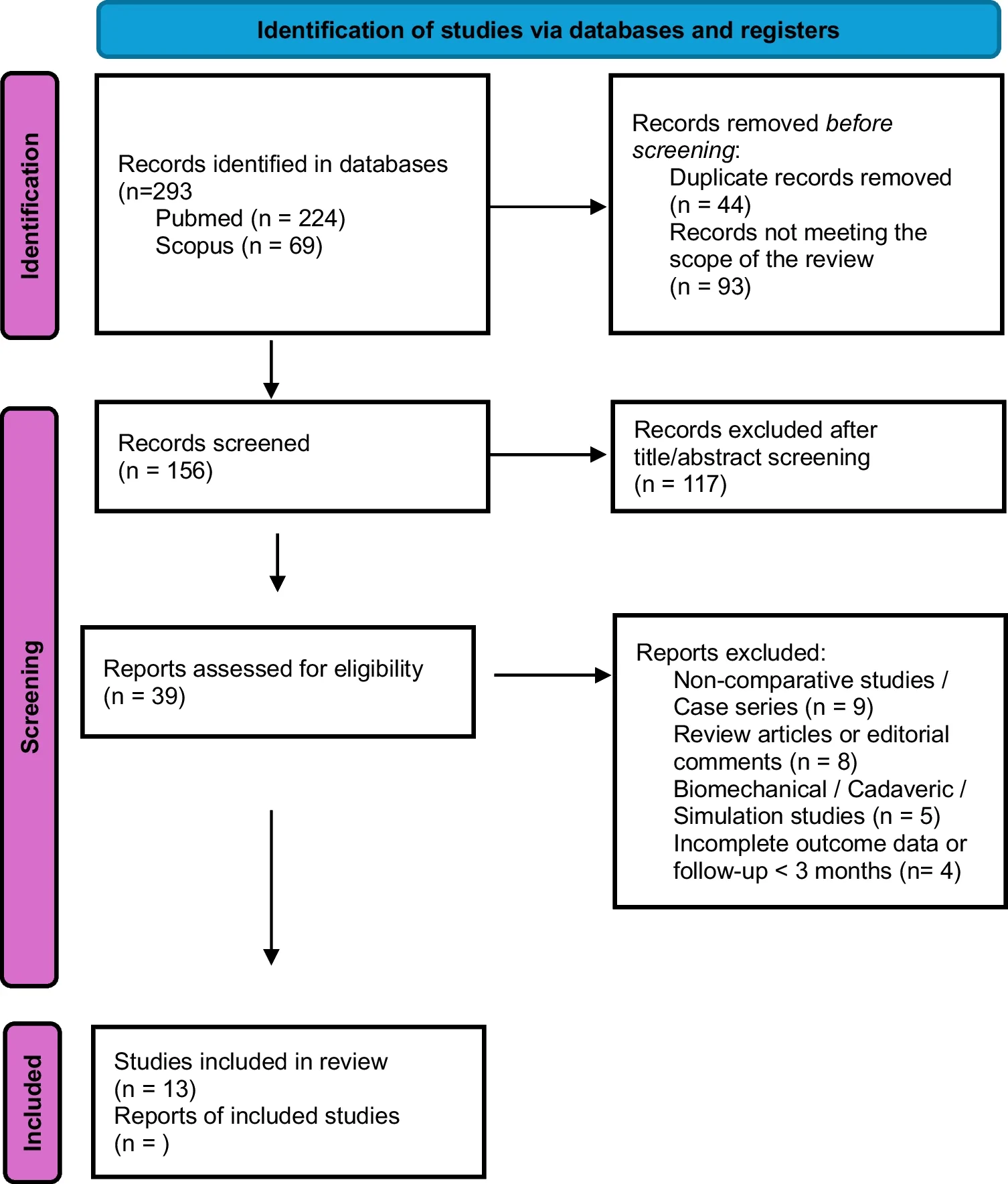
PRISMA 2020 flow diagram

The literature search was performed between September 7 and September 18, 2025. After duplicate removal, two reviewers independently screened titles and abstracts according to the predefined eligibility criteria. Potentially relevant articles underwent full-text review, and any disagreements were resolved by discussion until consensus was reached. The final selection was based on the predefined inclusion and exclusion criteria. The study selection process is summarized in Fig. 1. Ultimately, 13 studies were included in the qualitative synthesis and are summarized in Table 1.

**Table 1:** Selected articles

Author (Year)	Country	Study design	R-THA/C-THA (n)	Population/Indication	Robotic System	Main Results	Follow-up	Main Limitations	Methodological Quality
**Domb et al. (2020)** [8]	USA	Retrospective (PSM)	66/66	Primary Hip OA	MAKO®	Better functional outcomes, lower risk of placement outside safe zone, reduced LLD and overall offset discrepancy	> = 5 years	Single-surgeon series; small sample size to assess rare complications	MINORS: 22/24
**Guo et al. (2022)** [9]	China	RCT	45/48	Femoral head AVN or Hip Dysplasia	MAKO®	Reduced LLD and superior accuracy in acetabular cup positioning	3 months	Small sample size for safety endpoints; short follow-up period	RoB 2: Low risk
**Coulomb et al. (2023)** [10]	France	Retrospective (PSM)	98/98	Unilateral Hip OA (healthy contralateral hip)	MAKO®	Better functional outcomes, fewer complications, and superior radiological symmetry of center of rotation	12 months	Retrospective single-center design; non-standardized surgical approaches (anterior/posterior)	MINORS: 18/24
**Buchan et al. (2024)** [11]	USA	Retrospective cohort	107/105	Primary THA indications	ROSA®	Lower 90-day complications, shorter hospital stay, less pain at 2 weeks; no differences at 6 weeks	90 days	Radiographic placement not evaluated; single surgeon using direct anterior approach	MINORS: 20/24
**Di Gangi et al. (2024)** [12]	USA	Retrospective database (PSM)	1293/5873	Hip OA	MAKO®	Significantly lower dislocation rate (0.4% vs 1.2%) and fewer revisions for instability, especially in posterior approach	Mean 623 days	Large database study lacking detailed clinical/radiographic component placement data	MINORS: 18/24
**Lu et al. (2024)** [13]	China	Multicenter RCT	72/73	Primary THA indications	Trex-RS®	Reduced LLD and precise femoral stem alignment; no difference in acetabular orientation or function	6 months	Focus primarily on femoral stem placement; underpowered for rare complication detection	RoB 2: Low risk
**Tian et al. (2024)** [14]	China	Multicenter RCT	47/51	Hip OA and Developmental Dysplasia	JianJia®	Improved acetabular cup positioning; no significant differences in functional scores or LLD	3 months	Underpowered for complication assessment; short follow-up duration	RoB 2: Low risk
**Zhang S et al. (2024)** [15]	China	Retrospective (1:1 matching)	147/147	Developmental Dysplasia of the Hip	MAKO®	Greater cup accuracy in Crowe II–III dysplasia, reduced LLD, lower postoperative IL-6/CK; no functional differences	$\ge$6 months	Heterogeneous acetabular implants between groups; subgroup division by Crowe grade	MINORS: 18/24
**Howell et al. (2025)** [16]	USA	Retrospective database (PSM)	8033/8033	Primary THA indications	MAKO®	Lower 90-day revisit rates; reduced readmissions for dislocation, surgical site infection, and cellulitis	90 days	Industry database source (Stryker®); surgical approach and radiographic parameters omitted	MINORS: 19/24
**Shi et al. (2025)** [17]	China	Retrospective comparative	56/56	Developmental Dysplasia of the Hip	Longwell®	Improved safe-zone placement for cup and stem, reduced LLD, and better functional outcomes	12 months	Small sample size limiting statistical power for complication analysis	MINORS: 17/24
**Shah et al. (2025)** [18]	USA	Retrospective database (PSM)	2805/2805 (1 yr); 1143/1143 (5 yr)	Primary THA indications	Unspecified (TriNetX® base)	Lower risk of revision, dislocation, prosthetic pain, and periprosthetic femoral fracture at 5 years	Up to 5 years	Registry database limitations; lack of granular surgical approach and radiographic placement metrics	MINORS: 16/24
**Zhang J et al. (2025)** [19]	China	Retrospective (PSM)	74/74	Femoral neck fractures and Hip OA	JianJia®	Enhanced accuracy of component orientation; did not reduce subjective patient-perceived LLD	3 years	Small sample size to assess rare complications; single-region cohort	MINORS: 19/24
**Zhang X et al. (2025)** [20]	China	Secondary analysis of RCT	37/37	Primary THA indications (Ceramic-on-Ceramic)	Trex-RS®	Lower frequency of joint "squeaking", superior precision in center of rotation and cup placement; no functional differences	6 months	Small sample size; focused strictly on ceramic-on-ceramic bearing surfaces	RoB 2: Low risk

*OA* Osteoarthritis, *AVN* Avascular necrosis, *R-THA* Robotic Total Hip Arthroplasty (THA), Conventional-THA, *LLD* Leg Length Discrepancy, *IL* Interleukin, *CK* creatine kinase, *MINORS*: Methodological Index for Non-Randomized Studies, *PSM* Propensity Score Matching, *RCT* Randomized Controlled Trial, *RoB 2* Risk of Bias 2 tool, *R-THA* Robot-Assisted Total Hip Arthroplasty, *THA* Total Hip Arthroplasty; *USA*, United States of America

### Quality assessment

The methodological quality of the included studies was assessed according to study design. Randomized controlled trials were evaluated using the RoB 2 (Risk of Bias 2) tool, whereas non-randomized studies were assessed using the Methodological Index for Non-Randomized Studies (MINORS). Quality assessment focused on the risk of bias, methodological rigor, and overall study quality.

### Data synthesis

Because of the methodological heterogeneity of the included studies regarding study design, outcome measures, and follow-up duration, a quantitative meta-analysis was not considered appropriate. Therefore, the findings were synthesized qualitatively according to three predefined domains: (1) implant positioning accuracy, (2) functional outcomes, and (3) postoperative complications.

## Results

### Accuracy of implant positioning

A) Acetabular component positioning

Accurate acetabular component positioning is one of the primary objectives of robot-assisted total hip arthroplasty (R-THA), as it facilitates the restoration of hip biomechanics and may reduce the risk of instability, implant wear, and early failure. Most of the included studies assessed cup orientation according to predefined acetabular "safe zones", although the angular thresholds varied among studies. While the classical Lewinnek safe zone is defined as 40° ± 10° of inclination and 15° ± 10° of anteversion, other authors used alternative definitions, such as the Callanan criteria or study-specific target zones [21–23]. Overall, the available evidence is associated with greater accuracy of acetabular component positioning with R-THA compared with conventional THA (C-THA).

Domb et al. [8] reported significantly higher proportions of acetabular components positioned within both the Lewinnek (97.0% vs. 73.8%; *p* = 0.002) and Callanan (90.0% vs. 56.9%; *p* = 0.001) safe zones in the robotic group. Mean cup inclination (40.0° ± 3.2° vs. 43.0° ± 5.2°; *p* = 0.009) and anteversion (18.4° ± 3.7° vs. 16.3° ± 6.6°; *p* = 0.026) were also closer to the planned targets, with reduced variability. Similarly, Tian et al. [14] found that 80.9% of acetabular components in the robotic group were positioned within the predefined safe zone compared with 51.0% in the conventional group (*p* = 0.002). After adjustment for sex and body mass index, robot-assisted surgery remained independently associated with accurate cup placement (OR 4.09, 95% CI 1.62–10.37; *p* = 0.003).

In patients with developmental dysplasia of the hip, Zhang S et al. [15] also reported significantly greater accuracy with robotic assistance. Using a target orientation of 40° inclination and 20° anteversion (± 5°), the robotic technique achieved a higher proportion of cups within the target zone across all Crowe classifications, particularly in Crowe II–III deformities (84.3% vs. 56.9%; *p* = 0.002). Although Zhang J et al. [19] did not observe significant differences in the proportion of cups positioned within the Lewinnek safe zone (66.2% vs. 58.1%; *p* = 0.31), the robotic group was associated with significantly more accurate cup inclination (36.3° ± 7.6° vs. 40.0° ± 10.8°; *p* = 0.001) and anteversion (21.0° ± 7.5° vs. 23.9° ± 9.8°; *p* = 0.043).

Rather than evaluating angular safe zones, Coulomb et al. [10] assessed restoration of the hip centre of rotation (COR). Robotic surgery resulted in significantly fewer horizontal (> 5 mm) or vertical (> 3 mm) COR outliers than conventional THA (38.8% vs. 58.2%; *p* = 0.0099), with superior restoration of both horizontal and vertical COR coordinates (*p* < 0.007). Similar findings were reported in other comparative studies, all showing a consistent tendency toward more accurate cup orientation and greater adherence to predefined target zones with robotic assistance [13, 17, 20]. Although Guo et al. [9] found no significant differences in the proportion of cups located within the Lewinnek safe zone, robot-assisted THA was related to excellent agreement between planned and achieved component orientation (*p* < 0.001) (Table 2).

**Table 2:** Accuracy of implant positioning

Author (Year)	Cup Inclination (°)	p	Cup Anteversion (º)	p	Components within target zone (%)	p	*Global offset* (mm)	p	LLD (mm)	p
** Domb et al. 2020** (11)	40.0 ± 3.2 vs 42.6 ± 5.4	**0.009**	18.4 ± 3.7 vs 16.3 ± 6.6	**0.026**	97% vs 73.8% (Lewinnek safe zone)	**0.002**	3.0 ± 3.0 vs 4.5 ± 3.0	** < 0.001**	4.4 ± 3.5 vs 5.5 ± 4.1	0.091
** Guo et al. 2022** (12)	41.5 ± 4.2 vs 40.5 ± 6.4	0.338	21.1 ± 5.7 vs 17.3 ± 8.5	**0.006**	69.8% vs 64.4% (Lewinnek safe zone)	0.544	3.2 ± 1.3 vs 3.7 ± 1.6	0.311	3.2 ± 2.7 vs 6.9 ± 3.0	** < 0.001**
** Coulomb et al. 2023** (9)	-	-	-	-	Outliers 38.8% vs 58.2%	** < 0.01**	2.0 ± 6.4 vs 0.5 ± 7.7	0.145	1.1 ± 5.0 vs 0.3 ± 6.0	0.344
** Tian et al. 2024** (17)	[Δabs]* 3.45 ± 2.6 vs 5.71 ± 4.12	**0.002**	[Δabs] 6.33 ± 5.23 vs 12.01 ± 9.12	** < 0.001**	80.9% vs 51.0% (Lewinnek safe zone)	**0.002**	-	-	[Δabs] 0.89 ± 0.70 vs 0.96 ± 0.73	0.618
**Zhang S et al. 2024** Crowe (II/III) ** (20)	40.8 ± 3.4 vs 42.0 ± 5.3	0.176	21.4 ± 3.8 vs 24.3 ± 6.4	**0.007**	84.3% vs 56.9% (± 5° plan)	**0.002**	-	-	6.4 ± 4.1 vs 11.7 ± 8.2	**0.020**
**Lu X et al. 2024.** (15)	42.0 ± 6.7 vs 39.7 ± 4.0	0.065	20.21 ± 4.0 vs 21.7 ± 3.9	**0.047**	-	-	-	-	5.3 ± 4.3 vs 5.7 ± 5.4	0.686
** Shi et al., 2025** (14)	-	-	-	-	100% vs 85.7%	** < 0.05**	-	-	2 (1–3) vs 3 (2–5)	** < 0.001**
**Zhang J et al. 2025** (18)	36.3 (32.2–39.8) vs 40.0 (34.7–45.5)	**0.001**	21.0 ± 7.5 vs 23.9 ± 9.8	**0.043**	66.2% vs 58.1% (Lewinnek safe zone)	0.31	3.9 (3.5, 4.4) vs 5.3 (4.2, 5.8)	** < 0.001**	6.6(4.0–9.0) vs 9.8 (6.7–14.5)	** < 0.001**
**Zhang X et al. 2025** (19)	41.6 ± 6.2 vs 42.0 ± 7.9	0.806	17.5 ± 3.6 vs 18.3 ± 3.4	0.374	-	-	0.9 ± 0.7 vs 11.5 ± 10.9	** < 0.001**	2.8 ± 1.7 vs 6.2 ± 5.7	**0.001**

Values are presented as mean ± standard deviation or median (range/interquartile range), according to the reporting format of the original publication. Statistically significant p-values (p<0.05) are highlighted in **bold**

[Δabs] indicates the absolute error; the diff erence between the measured value and the planned value

**The study reported radiological outcomes separately according to Crowe classification. For consistency, only the Crowe II/III subgroup is presented in this table

B) *Limb length discrepancy*

Restoration of limb length is another important objective of THA, as discrepancies greater than 10 mm may lead to limping, gait disturbances, low back pain, and patient dissatisfaction [24]. Most of the included studies demonstrated improved radiographic restoration of limb length with robot-assisted THA.

Zhang J et al. [19] reported significantly lower postoperative limb length discrepancy (LLD) in the robotic group (6.6 ± 4.6 mm vs. 9.8 ± 7.8 mm; *p* < 0.001). However, subjective perception of LLD was comparable between groups, suggesting that radiographic improvement was not always clinically perceptible. Lu et al. [13] also observed significantly smaller postoperative LLD following robotic surgery (2.87 ± 1.55 mm vs. 5.81 ± 6.27 mm; *p* < 0.001). Although no significant differences were detected during subsequent follow-up, no patient in the robotic group presented an LLD greater than 10 mm, whereas 15.1% of patients in the conventional group exceeded this threshold. Comparable results were reported by Zhang X et al. [20] (2.8 ± 1.7 mm vs. 6.2 ± 5.7 mm; *p* = 0.001) and Guo et al. [9] (3.5 ± 2.7 mm vs. 6.9 ± 3.0 mm; *p* < 0.001). Likewise, Shi et al. [17] identified only one patient with LLD greater than 10 mm in the robotic cohort compared with twelve patients in the conventional group (*p* < 0.001). In contrast, Domb et al. [8], Coulomb et al. [10], and Tian et al. [14] found no statistically significant differences between techniques.

Overall, robot-assisted THA appears to improve the accuracy and consistency of limb length restoration, particularly by reducing the number of patients with clinically relevant discrepancies.

C) Femoral component positioning

Only one of the included studies provided a detailed evaluation of femoral component positioning. Lu et al. [13] reported significantly better coronal alignment of the femoral stem following robot-assisted THA (1.78° ± 0.64° vs. 2.22° ± 1.11°; *p* < 0.005), indicating more neutral stem positioning and less tendency toward varus or valgus alignment. Although no significant differences were observed in mean femoral anteversion (14.7° ± 8.9° vs. 12.9° ± 8.4°; *p* > 0.05), the robotic technique more consistently reproduced the planned femoral anteversion, suggesting improved accuracy of femoral component implantation.

### Functional outcomes

Functional outcomes were assessed using several patient-reported outcome measures (PROMs), including the Harris Hip Score (HHS), Oxford Hip Score (OHS), Western Ontario and McMaster Universities Osteoarthritis Index (WOMAC), Forgotten Joint Score-12 (FJS-12), and the Visual Analogue Scale (VAS). Follow-up ranged from the early postoperative period to more than five years, precluding direct quantitative comparison across studies. All studies reported substantial functional improvement after THA regardless of the surgical technique. Final HHS values generally ranged between 85 and 95 points, indicating good to excellent clinical outcomes in both robot-assisted and conventional THA.

Most comparative studies found no statistically significant differences in functional outcomes between techniques. Tian et al. [14] reported comparable HHS scores three months after surgery (89.9 ± 7 points; p > 0.05), while Zhang S et al. [15] observed no significant differences in either HHS (92.9 ± 9.8 vs. 91.4 ± 11.2; *p* = 0.22) or FJS-12 (78.3 ± 19.5 vs. 76.6 ± 22.7; *p* = 0.49). Similarly, Guo et al. [9], Lu et al. [13], Buchan et al. [11], and Zhang X et al. [20] found equivalent postoperative improvements in HHS, WOMAC, and VAS scores throughout follow-up.

Nevertheless, several studies reported modest functional advantages associated with robot-assisted THA. Coulomb et al. [10] found significantly higher FJS-12 (82.1 ± 22.0 vs. 71.2 ± 22.8; *p* < 0.05) and OHS (40.8 ± 0.8 vs. 38.1 ± 9.7; *p* < 0.05) scores one year after surgery, although HHS remained similar between groups. Shi et al. [17] also demonstrated superior postoperative HHS (86.6 ± 2.5 vs. 82.6 ± 2.5; *p* < 0.001) and FJS-12 scores (81.3 [79.2–83.9] vs. 79.1 [76.5–80.8]; *p* < 0.01) following robotic surgery. Longer-term follow-up by Domb et al. [8] suggested that these differences may persist over time, with significantly higher HHS and FJS-12 scores observed after more than five years (*p* < 0.01). An additional patient-centered outcome was reported by Zhang J et al. [19], who evaluated the Patient Acceptable Symptom State (PASS). A significantly greater proportion of patients undergoing robot-assisted THA achieved an acceptable symptomatic state compared with the conventional group (64.9% vs. 45.9%; *p* = 0.021). These patients also achieved higher postoperative HHS scores (96 vs. 92; *p* = 0.009).

Overall, although several studies reported statistically significant improvements in selected PROMs following robot-assisted THA, the majority found comparable functional recovery between robotic and conventional techniques. Current evidence therefore does not support a consistent or clinically meaningful functional superiority of robot-assisted THA during short- to mid-term follow-up.

### Complications

Postoperative complications were reported in all included studies, although event rates were generally low. Several investigations found no complications in either treatment group during follow-up, reflecting the overall safety of both robotic and conventional THA [9, 13–15, 17, 20].

The strongest evidence comes from Howell et al. [16], who analyzed more than 16,000 matched patients. Robot-assisted THA was linked to significantly lower rates of readmission for dislocation (0.09% vs. 0.39%; *p* < 0.001), surgical site infection (0.04% vs. 0.20%; *p* = 0.004), and cellulitis (0.01% vs. 0.10%; *p* = 0.039). In addition, the overall risk of 90-day readmission was reduced by 29% in the robotic group (OR 0.71, 95% CI 0.58–0.89; *p* = 0.002), without increasing emergency department visits or major medical complications. Similarly, Buchan et al. [11] reported significantly fewer postoperative complications within 90 days following robot-assisted THA (0.9% vs. 6.7%; *p* = 0.029), with no cases of postoperative dislocation or periprosthetic fracture in the robotic cohort.

Several studies also demonstrated lower revision rates after robotic surgery. Shah et al. [18] reported significantly fewer revision procedures at both one year (*p* < 0.001) and five years (*p* = 0.001), accompanied by a lower incidence of late dislocation and periprosthetic fracture. Likewise, Domb et al. [8] observed no reoperations or implant loosening after more than five years of follow-up in the robotic group, whereas three revision procedures were required following conventional THA because of component malposition or early polyethylene wear.

Coulomb et al. [10] found a significantly lower rate of minor postoperative complications in the robotic cohort (17.3% vs. 28.6%; *p* = 0.042). No revision procedures were required after robot-assisted THA, compared with a 5.1% revision rate following conventional surgery (*p* = 0.025), mainly because of instability or periprosthetic fracture. Interestingly, three postoperative dislocations occurred in the robotic group, whereas none were observed after conventional THA; however, all were successfully managed by closed reduction and the difference was not statistically significant (*p* = 0.114).

Di Gangi et al. [12] compared robot-assisted THA, computer navigation, and conventional surgery. Robot-assisted THA showed the lowest incidence of postoperative dislocation (0.4% vs. 1.2% with conventional THA; *p* = 0.008), while navigation produced intermediate results (0.9%). After adjustment for potential confounders, robotic surgery remained independently associated with a reduced risk of dislocation (OR 0.3, 95% CI 0.1–0.7; *p* < 0.007). Revision surgery (1.7% vs. 2.8%; *p* = 0.031) and overall surgical complications were also significantly lower in the robotic group. Subgroup analysis demonstrated that this protective effect was particularly evident in procedures performed through the posterior approach. Finally, Zhang J et al. [19] reported no cases of infection, dislocation, or periprosthetic fracture following robot-assisted THA during two years of follow-up, whereas two dislocations and one deep infection occurred after conventional surgery.

Overall, the available evidence suggests that robot-assisted THA is at least as safe as conventional THA and may reduce the incidence of selected postoperative complications, particularly dislocation, revision surgery, and early readmission. However, because most included studies reported relatively few adverse events, larger prospective studies with longer follow-up are required to confirm these findings.

## Discussion

The present systematic review demonstrates that robot-assisted total hip arthroplasty (R-THA) consistently improves the accuracy and reproducibility of implant positioning compared with conventional THA (C-THA). Across the included studies, robotic assistance resulted in more precise acetabular component orientation, better restoration of hip biomechanics, and fewer radiographic outliers. Nevertheless, these technical advantages were not consistently accompanied by superior functional outcomes. This discrepancy between radiological precision and clinically meaningful benefit represents the principal finding of the present review.

Interpretation of these results should consider the considerable heterogeneity of the available evidence. The included studies differed substantially regarding patient characteristics, preoperative diagnosis, robotic platform, surgical approaches, radiographic targets, outcome measures, and follow-up durations, limiting direct comparison between studies. The studies included in this review included five different robotic systems that are not comparable in terms of methodology (MAKO®, ROSA®, Trex-RS®, JianJia®, Longwell®, and Trex-RS®).

The most widely used systems, excluding China, are Mako® (Stryker®), and Rosa® (Zimmer-Biomet®), with Mako being the one most frequently reported in the literature [8–10, 12, 15, 16]. Both differ considerably in their workflows and levels of automation. MAKO®, is a CT-based robotic-assisted system while ROSA® is fluoroscopy-assisted [11]. The aim of this review was not to analyze the different robotic systems, but rather the use of robotics itself in primary THA. The goal of all of them is to achieve correct component positioning. The findings of this review should be interpreted as reflecting robot-assisted THA as a broad concept rather than the performance of any individual platform. Authors do not want to introduce an interpretive bias by presenting the advantages or disadvantages of each of the systems.

### Implant positioning accuracy and biomechanical restoration

Accurate implant positioning remains one of the principal advantages of robotic assistance. Most comparative studies demonstrated significantly greater adherence to predefined acetabular target zones and more consistent restoration of hip biomechanics following R-THA. Because component malposition has been associated with instability, impingement, accelerated polyethylene wear, and early revision, reducing radiographic outliers may represent one of the main clinical benefits of robotic technology. However, it should also be recognized that most studies provided limited information regarding preoperative planning and intraoperative guidance in the conventional THA group. Consequently, it remains uncertain whether the observed differences are attributable exclusively to robotic assistance or partially reflect differences in surgical planning and intraoperative verification.

Although the Lewinnek and Callanan safe zones remain useful reference benchmarks, contemporary hip arthroplasty increasingly recognizes that no universal safe zone guarantees optimal outcomes. Functional spinopelvic alignment, combined anteversion, surgical approach, soft-tissue balance, and patient-specific anatomy all influence hip stability. Indeed, recent evidence has shown that optimal acetabular cup orientation may differ according to the surgical approach, supporting the concept that implant positioning targets should be individualized rather than universally applied [23, 25]. In this context, the principal advantage of robotic assistance may lie not only in consistently achieving predefined targets but also in accurately executing patient-specific implant positioning strategies.

Although the available literature has focused primarily on acetabular component positioning, the femoral component should not be overlooked. Accurate restoration of femoral offset, stem alignment, femoral version, and limb length are essential to optimize hip biomechanics, abductor function, and joint stability [13, 26]. The limited evidence available suggests that robotic assistance may also improve femoral reconstruction, although considerably fewer studies have investigated this aspect.

### Radiographic precision versus patient perception

An intriguing observation emerging from the reviewed studies is that improved radiographic restoration does not necessarily translate into improved patient perception. Zhang J et al. demonstrated that despite significantly better restoration of limb length, subjective limb length discrepancy remained comparable between robotic and conventional THA. These findings suggest that restoration of radiographic parameters alone may be insufficient to predict patient satisfaction. Soft-tissue balance, spinopelvic mechanics, muscular adaptation, and patient expectations probably contribute substantially to postoperative function and satisfaction.

### Functional outcomes and measurement sensitivity

Functional outcomes remain one of the most controversial aspects of robotic THA. While all studies reported marked postoperative improvement regardless of surgical technique, most failed to demonstrate clinically meaningful differences in conventional outcome measures such as the Harris Hip Score (HSS). One possible explanation is the well-recognized ceiling effect of these instruments, as conventional THA already yields excellent clinical outcomes in most patients [8, 10, 13, 14]. More discriminative patient reported outcomes measures (PROMs), particularly the Forgotten Joint Score-12 (FJS-12), may better identify subtle differences between good and excellent outcomes. Indeed, several studies reported significantly higher FJS-12 scores following robotic surgery, although these findings were not consistently reproduced across all cohorts [27]. Furthermore, statistically significant differences should be interpreted in the context of their clinical relevance, as small improvements may not exceed established thresholds for meaningful patient benefit.

### Complications and surgical approach

Regarding postoperative complications, the available evidence suggests that R-THA is at least as safe as conventional THA and was associated with lower rates of selected mechanical complications, particularly dislocation and revision surgery. Several large registry studies and matched cohort analyses reported associations between robot-assisted THA and lower rates of postoperative instability, readmission, and revision. However, these findings derive predominantly from observational studies and should not be interpreted as establishing a causal relationship, since residual confounding, surgeon experience, institutional factors, and patient selection may have influenced the observed results. Therefore, although the current trend favours robotic assistance, definitive conclusions regarding complication reduction should be interpreted cautiously.

The interaction between robotic assistance and surgical approach also deserves consideration. Emerging evidence suggests that the potential benefit of robotics may vary according to the surgical approach employed. Some studies have reported a greater reduction in instability when robotic assistance is combined with the posterior approach, whereas others suggest that fluoroscopic guidance may play a more important role during direct anterior THA [28]. Consequently, the relative contribution of robotic technology, surgical approach, and surgeon experience remains incompletely understood.

### Learning curve, economic considerations, and literature alignment

Beyond clinical outcomes, several practical considerations influence the widespread adoption of robotic technology. Despite its potential benefits, there are some drawbacks to robotic-assisted THA. Robotic systems and their associated technologies can be expensive, potentially increasing the procedure cost [29]. Initial acquisition costs, maintenance expenses, increased operative time during the learning phase, the need for dedicated training, and, for some platforms as Mako®, additional preoperative CT imaging and disposable instrumentation continue to represent important barriers [30, 31]. However, this may vary depending on each country's healthcare system and the fee-for-service payment model. It will also vary depending on whether the hospital is public or private. Nevertheless, the available evidence suggests that the learning curve is relatively short, with operative times approaching those of conventional THA after approximately 12 to 17 procedures [12–15]. Moreover, some economic analyses have suggested that robotic THA may become cost-effective in high-volume centers through reductions in revision procedures and postoperative complications, although these findings may not be directly applicable to all healthcare systems [32]. Furthermore, the economic value of robotic THA is likely to depend on institutional case volume, local healthcare reimbursement models, and the specific robotic platform employed. The cost-effectiveness of robot-assisted THA will depend on the realization and long-term demonstration of its radiological and clinical benefits. Given the high costs associated with robotic systems—and the fact that manual THA is already a proven, successful procedure—wider adoption in their use, future developments and long-term patient follow-up will help determine whether it is cost-effective. Whether the benefit of robotic-assisted-THA outweighs its additional costs to payers and hospitals is also uncertain. The current literature is limited, with a paucity of cost effectiveness data and inconsistent findings regarding the outcomes of using robot assistance [4, 33].

The conclusions of the present review are consistent with previous systematic reviews and meta-analyses, which have consistently demonstrated improved implant positioning accuracy with robotic assistance but limited evidence of superior short-term functional outcomes [29, 33–35]. More recent reviews suggest that potential clinical benefits may become more evident with longer follow-up, particularly regarding patient-reported outcomes and implant survivorship [23]. However, definitive evidence remains unavailable [36–41]. Thus, a very recent study by Annapareddy et al. [40] compares functional outcomes and complication rates between robot-assisted and manual THA, with a minimum follow-up of three years. This is a retrospective observational cohort study that included consecutive patients undergoing primary THA at a single high-volume arthroplasty centre. The primary outcome was the Oxford Hip Score at three years. Secondary outcomes included modified HHS, Hip Disability and Osteoarthritis Outcome Score for Joint Replacement, FJS, and complications including dislocation, infection, readmission, and reoperation. Conclusions are that both robotic-assisted and manual THA provided excellent medium-term outcomes. Robotic-assisted THA demonstrated modest functional advantages and a trend toward lower complication rates, although these differences were not clinically meaningful. Furthermore, a very recent publication that evaluates the abstracts of systematic reviews on robotic-assisted THA, concludes that there was a high prevalence of selection bias in the abstracts of systematic literature reviews. The most common type of bias was the selective presentation of results favoring robotic-assisted THA [41].

Several limitations should be acknowledged. First, most included studies were observational, with relatively few randomized controlled trials available. Second, follow-up was generally limited to short- and medium-term outcomes, preventing assessment of long-term implant survivorship. Third, important methodological heterogeneity existed regarding robotic platforms, surgical approaches, radiographic targets, functional outcome measures, and patient populations. The included studies evaluated CT-based, fluoroscopy-assisted, and full-process robotic systems with different workflows and levels of automation. Consequently, the findings should be interpreted as reflecting robot-assisted THA as a broad concept rather than the performance of any individual platform. Fourth, many studies included relatively small cohorts, increasing the possibility of type II error for rare complications. Fifth, due to the rapid adoption of robotics in THA, new comparative studies published after the search completion date (September 2025) were not included in this synthesis. However, the findings of these recent publications do not alter the conclusions of this review [36–40]. Finally, potential industry involvement was not consistently reported, introducing the possibility of sponsorship bias.

### Future directions

Overall, current evidence supports the technical superiority of robot-assisted THA in terms of implant positioning accuracy and reproducibility. However, whether this improved precision translates into clinically meaningful benefits for the average patient remains uncertain. Future research should determine which patient populations benefit most from robotic assistance, particularly those with complex anatomy, spinopelvic abnormalities, or a higher risk of instability. Future multicenter randomized trials with standardized surgical protocols, longer follow-up, validated PROMs, and cost-effectiveness analyses are required to establish the true clinical value of robotic technology in primary THA. Furthermore, robot-assisted surgery for arthroplasty was first introduced for the knee [42]. A recent multicenter, participant- and assessor-masked study on Mako® robotic-arm-assisted total knee replacement (TKR) reached the same conclusion as this review on robotic-assisted THA: at twelve months post-randomization, robotic-assisted TKR did not provide a clinically meaningful benefit to patients [43].

## Conclusions

Robot-assisted total hip arthroplasty provides more accurate and reproducible implant positioning than conventional THA. Although robotic-THA is associated with lower rates of selected mechanical complications, particularly dislocation and revision surgery, current evidence does not demonstrate consistent, clinically meaningful improvements in functional outcomes. The principal value of robotic assistance may therefore lie in the reliable execution of individualized surgical plans rather than in improving average short-term functional outcomes. Long-term prospective studies are needed to determine whether these technical advantages translate into better implant survivorship and patient-reported outcomes.

## Data Availability

No datasets were generated or analysed during the current study.
